# Dataset of reptiles in fragmented forests at Tasik Kenyir, Hulu Terengganu, Malaysia

**DOI:** 10.1016/j.dib.2019.104994

**Published:** 2019-12-12

**Authors:** Siti Aisyah Komaruddin, Nurul Ain Mohamad, Muhamad Fatihah-Syafiq, Baizul Hafsyam Badli Sham, Mazrul Aswady Mamat, Nurulhuda Zakaria

**Affiliations:** Faculty of Science and Marine Environment, Universiti Malaysia Terengganu, 21030, Kuala Nerus, Terengganu, Malaysia

**Keywords:** Herpetofauna, Man-made lake, Conservation, Visual encounter survey, Drift-fenced pitfall trap

## Abstract

This data article is about reptiles (lizard, snake, and skink) captured from fragmented forest within man-made lake of Tasik Kenyir that is situated in Terengganu State, Peninsular Malaysia. Data collection was conducted in January 2019 and sampling methods included drift fenced-pitfall traps and Visual Encounter Survey (VES). All animals were identified, measured snout to vent (SVL) and weighted before their release at the site of capture. The highlights like conservation statuses in the wild, detection type and substrate type are presented with the data to increase its value. A total of 73 individuals from 18 species, 15 generas and seven families of reptiles were recorded. The data comprised of seven reptile family groups Agamidae, Gekkonidae, Scincidae, Colubridae, Elapidae, Viperidae and Homalopsidae. Reptiles like *Cyrtodactylus quadrivirgattus* (n = 33, 45.2%) and *Aphaniotis fusca* (n = 7, 9.6%) were most dominant in the checklist and most of the animals were captured using VES. Data of SVL and mass of the animals can be further interpreted by researchers to assess the health condition of animals in the altered habitats.

**Table:** Specifications Table

Subject	Agricultural and Biological Sciences
Specific subject area	Animal Science and Zoology
Type of data	Table
How data were acquired	Visual Encounter Survey (VES) and drift-fenced pitfall trap survey, Vernier caliper, (sensitivity 0.1 cm), measuring tape and analytical balance (sensitivity 0.1 kg)
Data format	Raw
Parameters for data collection	Data collection at various habitats such as streams, forest, roadside and man-made building. Survey conducted at both day and night to give equal temporal representation for both diurnal and nocturnal animal.
Description of data collection	Visual Encounter Survey (VES) involved active searching of animals for both day and night using wide-beam headlamp and torchlight. Passive survey using ‘L’ shaped drift-fenced pitfall trap constructed in the forest with at least 50 m spacing between each set of trap.
Data source location	Hulu Terengganu, Terengganu, East Peninsular Malaysia Pengkalan Utama: 5°09′01.0″N 102°44′56.2″E − 5°09′01.2″N 102°47′15.5″E
Data accessibility	With the article

**Table:** 

**Value of the Data** • The reptilian abundance data allows researchers to collaborate, extend their checklist, construct a repository and expand their statistical analyses. • The abundance data along with their morphological descriptions also informs about the variety of reptiles present in sub-urban area and fragmented forest within man-made lake that offer research opportunities and perhaps collaboration to address the subject matter. • The size and weight data of reptiles can be translated into body condition index by the researcher and can be integrated into indicator of wildlife health, stress and forest carrying capacity. • Detection type and substrate type data can be incorporated into prediction models that can be used in planning conservational management by responsible authorities. Substrate type data also can address specific microhabitat associations of reptiles that may be used to illustrate evolutionary processes of speciation by taxonomist.

## 1 Data

The data was constructed using 73 reptilian individuals sighted at various habitats within Tasik Kenyir. In this six days of passive and active samplings, a total of 18 reptilian species from seven family groups were transformed into Table 1 to indicate abundances. Additional information like common name and conservation statuses acquired from IUCN Red List were compiled with the data to focus valuable reptilian species available in Kenyir rainforest. Total family and individual counts were presented in Table 2. Complete raw data on reptiles capture along with the capture date, detection type, substrate type and additional morphological descriptions are available in a separate list (Table 3).

**Table 1:** Taxonomic classification, abundance and statuses in the wild of reptiles discovered from the sampling sites within Tasik Kenyir.

FAMILY/SPECIES NAME	COMMON NAME	NUMBER OF INDIVIDUAL	RELATIVE ABUNDANCE (%)	STATUS (IUCN)
AGAMIDAE				
*Aphaniotis fusca*	Earless agamid	7	9.6	LC
*Draco melanopogon*	Black-bearded gliding lizard	4	5.5	NE
GEKKONIDAE				
*Cnemaspis argus*	Lawit mountain rock gecko	4	5.5	LC
*Cyrtodactylus consobrinus*	Peter's forest gecko	4	5.5	LC
*Cyrtodactylus quadrivirgatus*	Marbled bent-toed gecko	33	45.2	LC
*Gekko monarchus*	Spotted-house gecko	3	4.1	NE
*Gekko smithii*	Large forest gecko	3	4.1	LC
*Hemidactylus frenatus*	Spiny-tailed gecko	4	5.5	LC
*Gehyra mutilata*	Four-clawed Gecko	1	1.4	NE
SCINCIDAE				
*Eutropis longicaudata*	Long-tailed sun skink	1	1.4	NE
*Eutropis multifasciata*	Many-lined sun skink	1	1.4	NE
COLUBRIDAE				
*Ahaetulla prasina*	Oriental whip snake	1	1.4	LC
*Boiga dendrophila*	Gold-ringed cat snake	1	1.4	NT
*Dendrelaphis pictus*	Painted bronzeback	1	1.4	NE
*Coelognathus flavolineatus*	Malayan racer	2	2.7	LC
ELAPIDAE				
*Bungarus flaviceps*	Red-headed krait	1	1.4	LC
VIPERIDAE				
*Parias hageni*	Hagen's pit viper	1	1.4	LC
HOMALOPSIDAE				
*Homalopsis buccata*	Puff-faced water snake	1	1.4	LC

Note: Conservation status of reptiles follow International Union for Conservation of Nature Red List (IUCN) descriptions whereby LC = Least Concern, NT = Near Threatened, and EN = Endangered.

**Table 2:** Family and total individual counts of reptiles at Tasik Kenyir.

FAMILY	NUMBER OF INDIVIDUAL
Agamidae	11
Gekkonidae	52
Scincidae	2
Colubridae	6
Homalopsidae	1
Elapidae	1
Viperidae	1

**Table 3:** The unprocessed data of reptiles captured within Tasik Kenyir rainforest.

DATE	SPECIES NAME	DETECTION TYPE	SUBSTRATE	SVL (mm)	WEIGHT (g)
2 January 2019	*Aphaniotis fusca*	C	V	5.7	4.5
	*Aphaniotis fusca*	C	V	5.5	4.3
	*Cyrtodactylus quadrivirgatus*	C	V	6.3	4.6
	*Cyrtodactylus quadrivirgatus*	C	L	4	3.4
	*Cyrtodactylus quadrivirgatus*	C	V	4.5	1.7
	*Cyrtodactylus quadrivirgatus*	C	V	6.9	6
	*Cyrtodactylus quadrivirgatus*	C	L	3.2	0.6
	*Cyrtodactylus quadrivirgatus*	C	L	4.2	1.2
	*Cyrtodactylus quadrivirgatus*	C	L	3.3	0.7
	*Gehyra mutilata*	C	L	2.5	0.3
	*Hemidactylus frenatus*	C	V	4.6	3.8
	*Ahaetulla prasina*	C	V	47.9	10.5
	*Coelognathus flavolineatus*	V	–	–	–
	*Cyrtodactylus consobrinus*	V	–	–	–
3 January 2019	*Dendrelaphis pictus*	C	V	66.5	68.8
	*Cyrtodactylus consobrinus*	C	R	6.7	5.2
	*Aphaniotis fusca*	C	V	4.2	1.4
	*Cyrtodactylus quadrivirgatus*	C	V	4.2	1.3
	*Cyrtodactylus quadrivirgatus*	V	V	–	–
	*Cyrtodactylus quadrivirgatus*	V	R	–	–
	*Cyrtodactylus quadrivirgatus*	V	R	–	–
	*Cyrtodactylus quadrivirgatus*	V	R	–	–
	*Gekko monarchus*	V	R	–	–
	*Gekko monarchus*	V	R	–	–
	*Gekko monarchus*	V	L	–	–
	*Parias hageni*	V	V	–	–
	*Draco melanopogon*	V	L	–	–
	*Draco melanopogon*	V	L	–	–
	*Draco melanopogon*	V	L	–	–
4 January 2019	*Draco melanopogon*	C	L	7.7	3.3
	*Aphaniotis fusca*	C	V	6.1	4.6
	*Hemidactylus frenatus*	C	T	3.1	0.6
	*Hemidactylus frenatus*	C	T	5	2.3
	*Cyrtodactylus quadrivirgatus*	C	V	5.9	3.2
	*Cyrtodactylus quadrivirgatus*	V	–	–	–
	*Cyrtodactylus quadrivirgatus*	V	–	–	–
	*Cyrtodactylus consobrinus*	V	–	–	–
	*Eutropis multifasciata*	V	–	–	–
	*Gekko smithii*	V	–	–	–
	*Gekko smithii*	V	–	–	–
5 January 2019	*Cnemaspis argus*	C	R	6.1	4.4
	*Gekko monarchus*	C	R	4.7	1.9
	*Aphaniotis fusca*	C	V	2.8	0.4
	*Cyrtodactylus quadrivirgatus*	C	V	6.7	4.8
	*Cyrtodactylus quadrivirgatus*	C	V	5.2	3
	*Cyrtodactylus quadrivirgatus*	C	V	7.2	6.6
	*Cyrtodactylus quadrivirgatus*	C	V	6	5.8
	*Cyrtodactylus quadrivirgatus*	C	V	6	4.2
	*Cyrtodactylus quadrivirgatus*	C	V	4	2
6 January 2019	*Cyrtodactylus quadrivirgatus*	C	V	6.6	5.9
	*Cyrtodactylus quadrivirgatus*	C	V	5.7	4.4
	*Cyrtodactylus quadrivirgatus*	C	V	5.6	4
	*Cyrtodactylus quadrivirgatus*	C	V	6.5	5.5
	*Cyrtodactylus quadrivirgatus*	C	V	6	4.4
	*Cyrtodactylus quadrivirgatus*	C	V	3.5	1.1
	*Cyrtodactylus quadrivirgatus*	C	V	6	2.4
	*Cyrtodactylus quadrivirgatus*	C	V	6.1	5.6
	*Cyrtodactylus quadrivirgatus*	C	V	6.7	6.3
	*Cyrtodactylus quadrivirgatus*	C	V	5.2	3.2
	*Cyrtodactylus quadrivirgatus*	C	V	5	2.3
	*Cyrtodactylus quadrivirgatus*	C	V	4.7	2.3
	*Cyrtodactylus quadrivirgatus*	V	V	–	–
	*Gekko monarchus*	C	R	6.7	6.3
	*Eutropis longicaudata*	C	X	7.6	13.9
	*Aphaniotis fusca*	C	V	4	1.6
	*Aphaniotis fusca*	C	V	2.3	0.5
	*Hemidactylus frenatus*	C	R	5.9	3.9
	*Cyrtodactylus consobrinus*	C	R	11.9	29.8
	*Cnemaspis argus*	C	R	5.3	3.9
	*Cnemaspis argus*	C	R	5.6	3.9
	*Cnemaspis argus*	C	R	5.6	3.6
	*Gekko smithii*	C	T	15.1	73.6
	*Boiga dendrophila*	C	V	51.2	19.3
	*Coelognathus flavolineatus*	C	R	127	379.4
	*Homalopsis buccata*	C	W	23.5	7.2

Note: Detection type are denote with V = visual observation and C = capture. Substrate type are denote with L = log, R = rock, T = building wall, V = vegetation, W = water, X = leaf litter.

Description of measurement is abbreviated as SVL = snout to vent length. Measurements are denote with g = gram and mm = millimeter.

## 2 Experimental design, materials, and methods

Animals were captured using drift fenced-pitfall trap which is known as passive sampling, and Visual Encounter Survey (VES) which is known as active sampling. These sampling methods follow the first reptile study that was conducted at Tasik Kenyir by Ref. [1]. The coupled techniques are necessary as drift fenced-pitfall trap alone only catch ground dweller reptiles and almost impossible to catch arboreal reptiles. Hence, VES is needed to capture both ground dwellers including aquatic reptiles and arboreal reptiles at the same time.

**Fig. 1:**
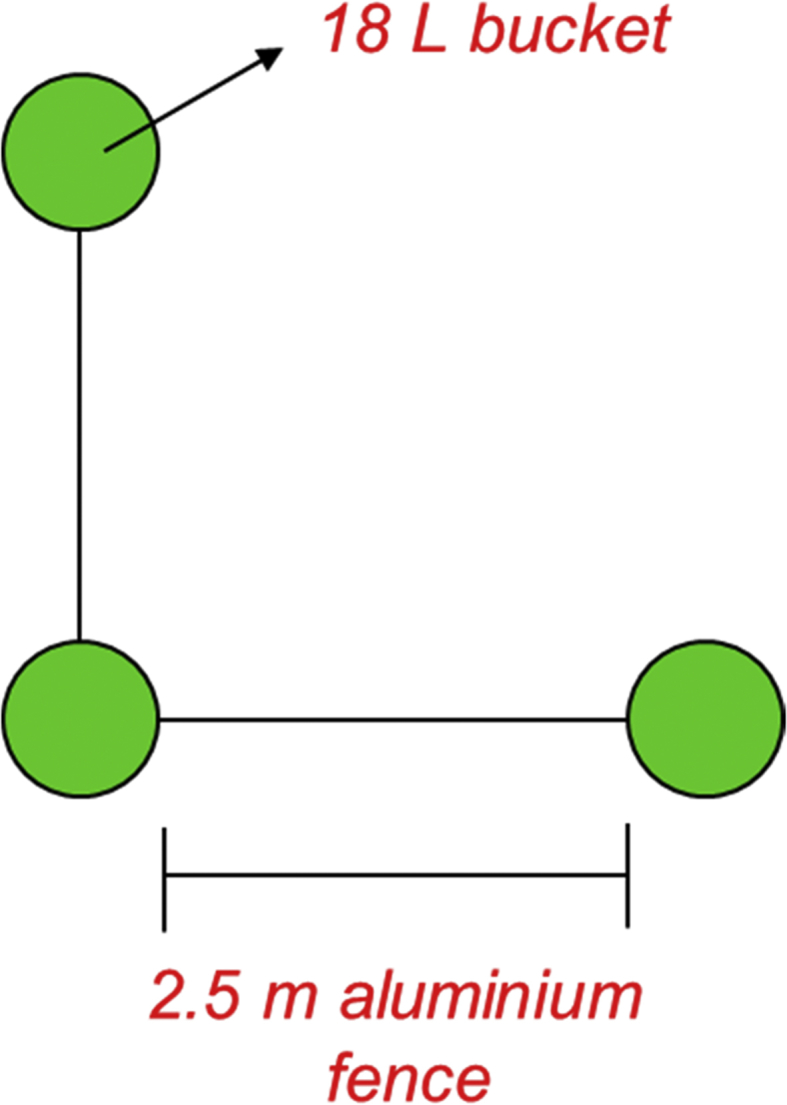
Illustration of a set of ‘L’ shape drift fenced-pitfall trap.

Drift fences are borders that used to guide the movement of terrestrial animals towards the traps. Pitfall traps were constructed from 18 L plastic bucket that were punched with small hole to allow the flowing of excess water and were buried into the ground up to its lid level. The aluminium sheets were used to separate the traps with distance of 2.5 m from each trap. Due to the geographical limitation, traps were set up in ‘L’ shape arrangement with the total of 12 traps and three traps for each set (Fig. 1). The arrays were replicated over multiple sites to determine the variation between different pitfall traps. Traps then were checked regularly before noon and also during the VES survey at night. VES involves active searching of animals for both day (1000H–1300H) and night (2000H–2300H). Day and night survey give an equal temporal representation to capture both diurnal and nocturnal species. During night time, a wide-beam headlamp or torchlight was used to discover animals in the dark by depending on the reflection produced by the animal's eyes.

Specimens were properly handled and transferred into different plastic bags to avoid them from having stress and injuries. Each of the specimen was identified by referring to various identification books such as [[2], [3], [4], [5]]. The photograph of each specimen was taken using a compact camera which included the dorsal and ventral sides. Measurements such as snout-vent length (SVL) was obtained using Vernier caliper while the weight was measured using an electronic balance. Voucher specimens of species collected were preserved in 10% formalin and later stored in 70% alcohol before deposited at Makmal Biologi Umum (FSSM, UMT) for future reference.

## References

[1] Zakaria A.A., Rahim N.A.A., Ahmad A.B., Abdullah M.T., Abdullah M.T., Mohammad A., Nor Zalipah M., Safiih Lola M. (2019). Species richness estimation of reptiles in selected sites of Tasik Kenyir, Hulu Terengganu, Malaysia. Greater Kenyir Landscapes.

[2] Berry P.Y. (1975). The Amphibian Fauna of Peninsular Malaysia.

[3] Das I. (2002). An Introduction to the Amphibians and Reptiles of Tropical Asia.

[4] Stuebing R.B., Inger R.F. (1999). A Field Guide to the Snakes of Borneo.

[5] Norhayati A. (2017). Frogs and Toads of Malaysia: Malaysia Biodiversity Information System (MyBIS).

